# Osteopontin Expression in Cardiomyocytes Is Increased in Pediatric Patients With Sepsis or Pneumonia

**DOI:** 10.3389/fphys.2018.01779

**Published:** 2018-12-10

**Authors:** Camila Iansen Irion, Kiera Parrish, Krista John-Williams, Sakir H. Gultekin, Lina A. Shehadeh

**Affiliations:** ^1^Interdisciplinary Stem Cell Institute, University of Miami Leonard M. Miller School of Medicine, Miami, FL, United States; ^2^Division of Cardiology, Department of Medicine, University of Miami Leonard M. Miller School of Medicine, Miami, FL, United States; ^3^Department of Pathology, University of Miami Leonard M. Miller School of Medicine, Miami, FL, United States; ^4^Vascular Biology Institute, University of Miami Leonard M. Miller School of Medicine, Miami, FL, United States; ^5^Peggy and Harold Katz Family Drug Discovery Center, University of Miami Leonard M. Miller School of Medicine, Miami, FL, United States

**Keywords:** osteopontin, sepsis, pneumonia, pediatric, heart, cardiomyocyte

## Abstract

Sepsis and pneumonia are major causes of death in the United States, and their pathophysiology includes infection with inflammation and immune dysfunction. Both sepsis and pneumonia cause cardiovascular dysfunction. The expression of Osteopontin (OPN) in cardiomyocytes of patients with sepsis or pneumonia, and its role the induced cardiac dysfunction have not been thoroughly investigated. OPN is a matricellular protein synthesized by multiple diseased tissues and cells including cardiomyocytes. Here, we studied the expression of OPN protein using immunofluorescence in human myocardial autopsy tissues from pediatric and mid age or elderly patients with sepsis and/or pneumonia. Fourteen human myocardial tissues from six pediatric patients and eight mid-age or elderly patients were studied. Immunofluorescence was used to investigate the expression of OPN in paraffin-embedded heart sections co-stained with the myocyte markers Actin Alpha 1 (ACTA1) and Myosin Light Chain 2 (MLC2). A quantitative analysis was performed to determine the number of ACTA1 and MLC2 positive cardiomyocytes that express OPN. The results showed that OPN expression was significantly increased in cardiomyocytes in the hearts from pediatric patients with sepsis and/or pneumonia (*N* = 3) relative to pediatric patients without sepsis/pneumonia (*N* = 3), or adult to elderly patients with sepsis/pneumonia (*N* = 5). Among the older septic hearts, higher levels of cardiomyocyte OPN expression was seen only in conjunction with severe coronary arterial occlusion. This is the first study to document increased OPN expression in cardiomyocytes of pediatric subjects with sepsis or pneumonia. Our findings highlight a potentially important role for OPN in sepsis- or pneumonia-mediated cardiac dysfunction in pediatric patients.

## Introduction

Osteopontin (OPN) is a matricellular protein synthesized by a range of tissues and cell types including immunological cells (macrophages and T cells) (Lund et al., 2009), fibroblasts (Ashizawa et al., 1996; Jin et al., 2011), epithelial cells (Kato et al., 2014), osteoclasts, and osteoblasts (Qing et al., 2014), vascular smooth muscle cells (Sodhi et al., 2001), renal tubular epithelial cells (Zhang et al., 2010; Cobbs et al., 2018; Ding et al., 2018), and cardiomyocytes (Ashizawa et al., 1996; Lorenzen et al., 2015). OPN has been implicated as an important regulator of inflammation, biomineralization, cellular viability, cancer, diabetes, and renal disease (Icer and Gezmen-Karadag, 2018), and in the last few years has been getting increasing attention in the cardiovascular field (Singh et al., 2014; Li et al., 2017; Icer and Gezmen-Karadag, 2018). Under normal physiological conditions, the heart expresses low levels of OPN (Singh et al., 2014). However, OPN expression in the heart increases significantly under pathophysiological states such as myocardial infarction, hypertrophy, and heart failure in both pre-clinical (Graf et al., 1997; Trueblood et al., 2001; Li et al., 2017), and clinical studies (Graf et al., 1997; Stawowy et al., 2002; Lopez et al., 2013).

Two important conditions, sepsis (Merx and Weber, 2007; Rudiger and Singer, 2007, 2013; Hunter and Doddi, 2010) and pneumonia (Corrales-Medina et al., 2011, 2012; Singanayagam et al., 2012; Brown et al., 2014) can compromise the heart causing myocardial dysfunction. Sepsis is a systemic inflammatory response to an infection and can lead to death due to multiple organ failure, including the organs of the cardiovascular system (Hunter and Doddi, 2010). In the heart, some of the sepsis-caused alterations include contractile systolic and diastolic dysfunction (Poelaert et al., 1997; Jardin et al., 1999; Landesberg et al., 2012), arrhythmias (Leibovici et al., 2007), and right heart failure (Dhainaut et al., 1988; Parker et al., 1990). The mechanisms responsible for sepsis-caused cardiac dysfunction could be multifactorial (Merx and Weber, 2007; Liu et al., 2017). Studies have suggested roles in sepsis-caused cardiac dysfunction for endotoxins released by lysis of gram-negative bacteria together with the presence of Toll-like receptor-4 and CD14 (Suffredini et al., 1989; Tavener et al., 2004; Rudiger and Singer, 2007), inflammatory cytokines (mainly TNF-α, IL-1) (Vincent et al., 1992; Kumar et al., 1996; Hunter and Doddi, 2010), nitric oxide (Dal-Secco et al., 2017), high level of circulating catecholamine (Bocking et al., 1979; Rudiger and Singer, 2007), endothelin-1 (Shindo et al., 1998), mitochondrial dysfunction, and oxidative stress (Khadour et al., 2002; Watts et al., 2004; Matkovich et al., 2017), or altered intracellular calcium trafficking (Zhong et al., 1997; Rudiger and Singer, 2013). Few studies reported the elevated serum levels of OPN in patients with sepsis (Vaschetto et al., 2008) or the association of OPN with inflammation and mortality in a mouse model of sepsis (Fortis et al., 2015). However, the expression of OPN in cardiomyocytes of septic patients, and its role in sepsis-mediated cardiac dysfunction have not been studied.

Pneumonia is an inflammatory disease of the lung caused by bacteria, viruses, or fungi, and it is responsible for high morbidity and mortality in adults and children (Mattila et al., 2014; le Roux and Zar, 2017). Some reports have shown that pneumococcal pneumonia can lead to cardiac complications including heart failure, arrhythmias, and myocardial infarction (Musher et al., 2007; Corrales-Medina et al., 2012). Besides these studies on increased levels of OPN in the plasma of patients with interstitial (Kadota et al., 2005) and community-acquired pneumonia (Chang et al., 2016), and in the plasma and lung of mice infected with *Streptococcus pneumonia* (van der Windt et al., 2011), the cardiomyocyte expression of OPN and its role in pneumonia-mediated cardiac dysfunction have not been investigated.

This study quantifies the expression of OPN protein using immunofluorescence in human myocardial autopsy tissues from pediatric and elderly patients with sepsis or pneumonia.

## Materials and Methods

### Human Myocardial Tissue Samples

A total of 14 human myocardial and two control kidney autopsy tissues were obtained from the Department of Pathology at the University of Miami Leonard M. Miller School of Medicine. The myocardial tissues belonged to six pediatric (<2 years old) patients and eight adult or elderly (48–77 years old) patients. Table 1 lists demographic data for autopsy cases used for this study. These kidney tissues (especially the diseased one) were used as positive controls for OPN expression as it is known that renal tubular cells express high levels of OPN under pathological conditions (Ding et al., 2018).

**Table 1:** Characteristics from the patient samples.

Sample ID	Age	Medical condition	Organ
A	≤2 years old	Enlarged right kidney due to left kidney agenesis	Right Kidney (Positive control for OPN staining)
H6	48–77 years old	Normal kidney	Kidney (Positive control for OPN staining)
H4	≤2 years old	The heart muscle was edematous due to sepsis and multi-organ failure. The baby was premature, developed a bacterial sepsis followed by necrotizing enterocolitis and aspiration pneumonia	Heart
G	≤2 years old	Acute lymphoblastic leukemia. Fungal pneumonia; Invasive pulmonary aspergillosis	Heart
H	48–77 years old	Septic shock; Left anterior descending artery (LAD) occluded >75%; Right circumflex artery occluded 100%; minimum fibrosis; no significant old infarct	Heart
H13	≤2 years old	The baby was premature, developed sepsis followed by necrotizing enterocolitis midgut volvulus	Heart
H9	48–77 years old	Deep venous thrombosis of pulmonary embolism. No abnormal histology of the heart	Heart
H3	48–77 years old	Left ventricular hypertrophy and papillary muscle infarct. Sepsis. Pericarditis	Heart
H1	48–77 years old	Pneumonia and colitis. Septic shock	Heart
H12	48–77 years old	Deep vein thrombosis of mesenteric artery embolism. No abnormal histology of the heart	Heart
D	≤2 years old	Full term ompholocele. Unremarkable myocardium	Heart
H7	48–77 years old	Bronchopneumonia. No abnormal histology of the heart	Heart
H10	≤2 years old	Premature, stillborn. Placentae abrubption. No abnormal histology of the heart	Heart
H5	48–77 years old	Colonic diverticulitis with perforation, sepsis. No abnormal histology of the heart	Heart
H11	≤2 years old	Premature. Holoprosencephaly. No abnormal histology of the heart	Heart
H2	48–77 years old	Hypertension; Sepsis; No abnormal histology of the heart	Heart


*Samples are listed in the order of highest to lowest OPN expression in the cardiomyocytes in 14 heart autopsy samples and in the renal tubules in two kidney autopsy samples used as controls for OPN staining.*

This study was carried out in accordance with the recommendations of the University of Miami Institutional Review Board (IRB protocol # 20180439). All autopsy specimens were de-identified and there was no need for consent forms.

### Staining and Image Acquisition

**FIGURE 1:**
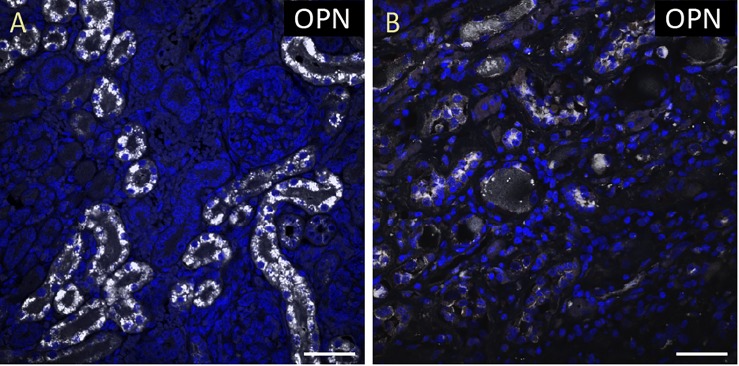
OPN is highly expressed in pediatric diseased kidney. **(A)** In the pediatric patient with enlarged right kidney, OPN (white) is highly expressed in the renal tubular epithelial cells. **(B)** In an elderly patient with a normal kidney, OPN (white) is moderately expressed in the renal tubular epithelial cells. Scale bar = 50 μm.

**FIGURE 2:**
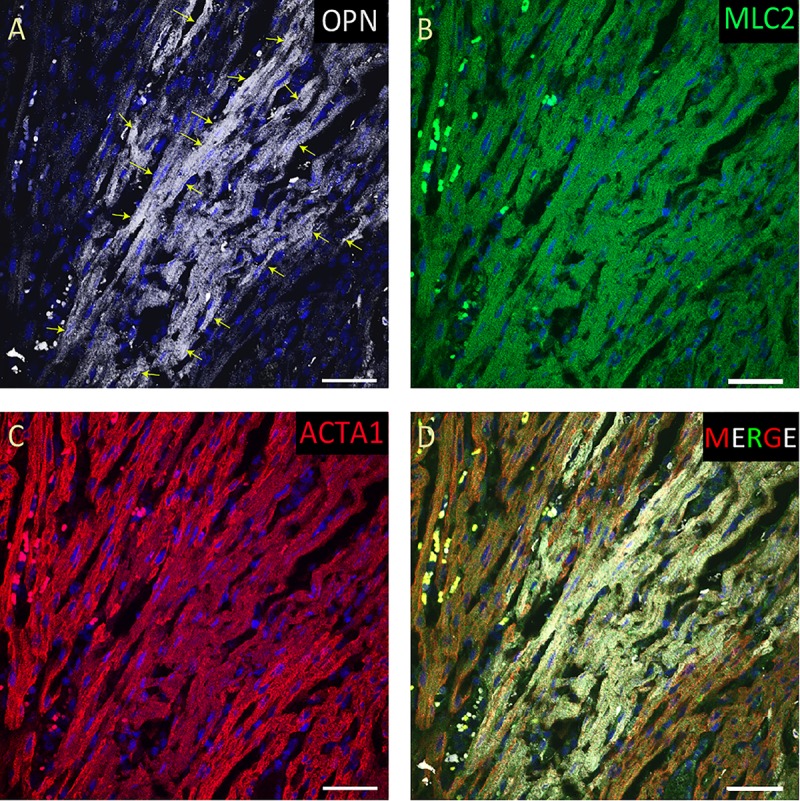
OPN expression is high in the cardiomyocytes from the pediatric patient with sepsis and pneumonia. In the pediatric patient with sepsis and pneumonia, OPN (white in **A**) is highly expressed in MLC (green in **B**), and ACTA1 (red in **C**) positive cardiomyocytes (arrows). **(D)** is the merged panel. Scale bar = 50 μm.

**FIGURE 3:**
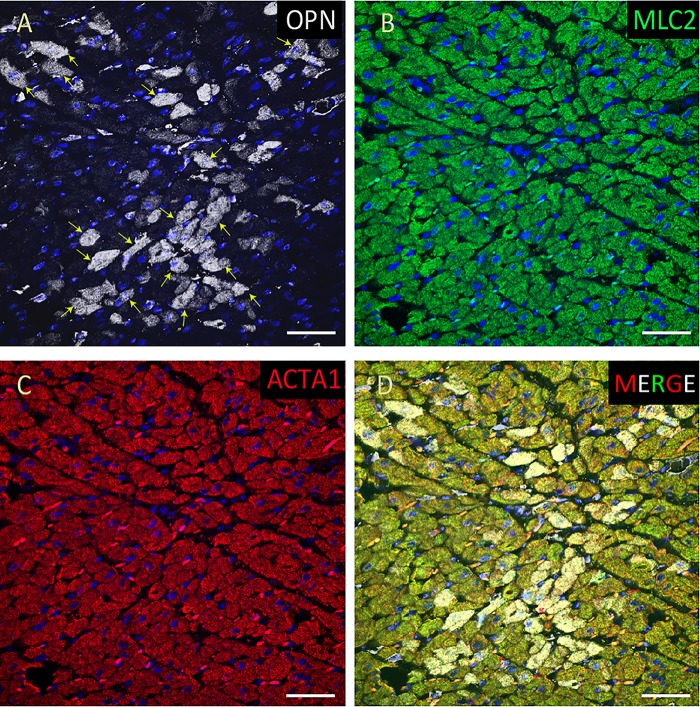
OPN expression is high in the cardiomyocytes from the pediatric patient with fungal pneumonia. In the pediatric patient with fungal pneumonia, OPN (white in **A**) is highly expressed in MLC (green in **B**), and ACTA1 (red in **C**) positive cardiomyocytes (arrows). **(D)** is the merged panel. Scale bar = 50 μm.

**FIGURE 4:**
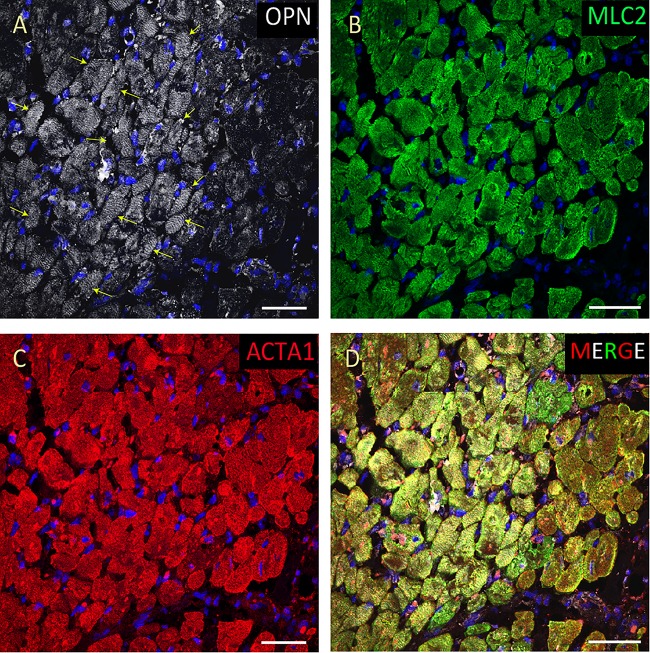
OPN expression is increased in the cardiomyocytes from the elderly patient with septic shock and coronary artery disease. In the elderly patient with septic shock and >75% occlusion in the left anterior descending (LAD) artery and 100% occlusion in the right circumflex artery (RCA), OPN (white in **A**) is highly expressed in MLC (green in **B**), and ACTA1 (red in **C**) positive cardiomyocytes (arrows). **(D)** is the merged panel. Scale bar = 50 μm.

**FIGURE 5:**
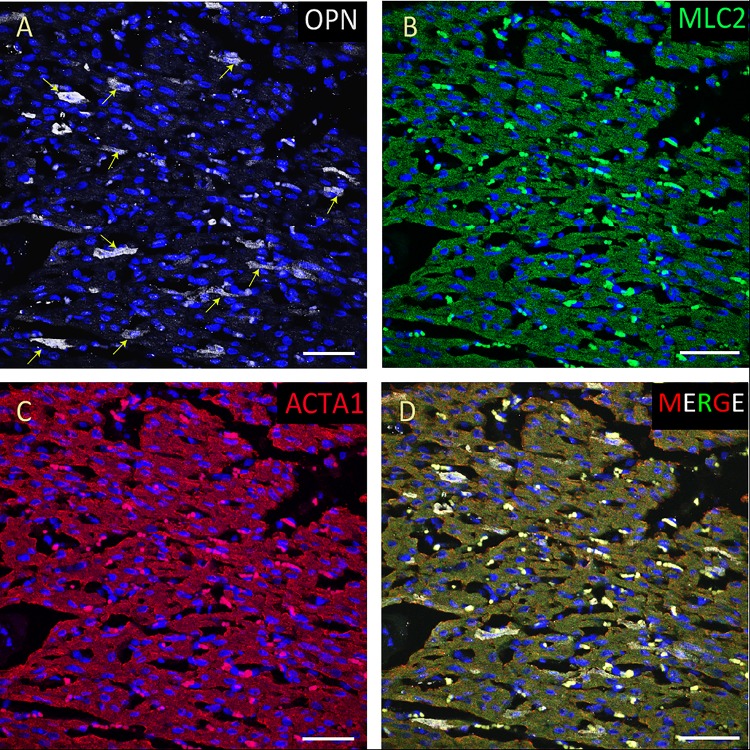
OPN expression is high in the cardiomyocytes from the pediatric patient with sepsis. In the pediatric patient with sepsis, OPN (white in **A**) is highly expressed in MLC (green in **B**), and ACTA1 (red in **C**) positive cardiomyocytes (arrows). **(D)** is the merged panel. Scale bar = 50 μm.

**FIGURE 6:**
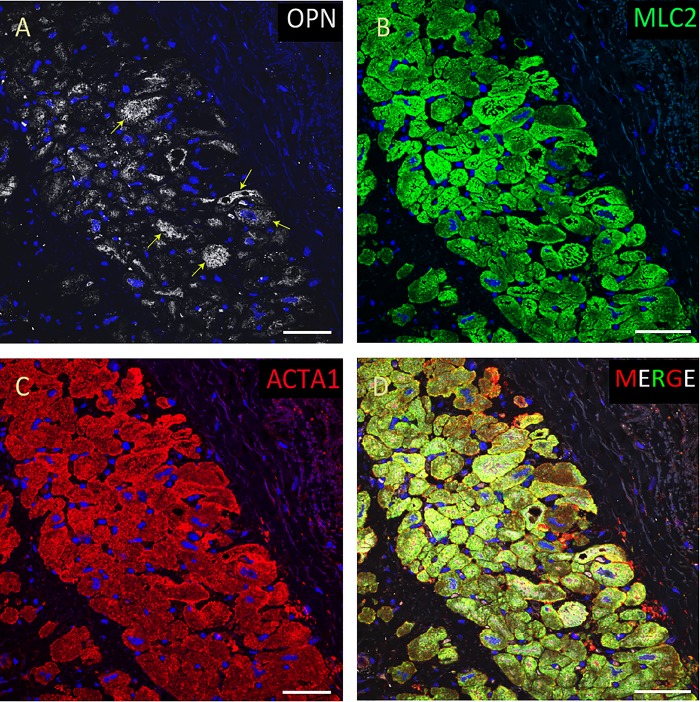
OPN expression is minimal in the cardiomyocytes from an elderly patient with left ventricular hypertrophy and papillary muscle infarct. In the elderly patient with left ventricular hypertrophy and papillary muscle infarct, OPN (white in **A**) is expressed in only a few number of MLC (green in **C**), and ACTA1 (red in **C**) positive cardiomyocytes (arrows). **(D)** is the merged panel. Scale bar = 50 μm.

**FIGURE 7:**
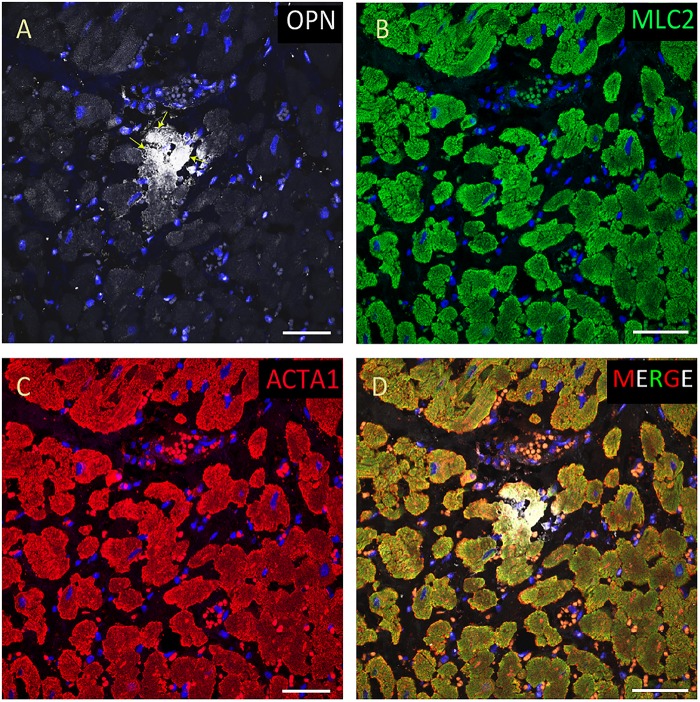
OPN expression is minimal in the cardiomyocytes from an elderly patient. In the elderly patient with sepsis and pneumonia, OPN (white in **A**) is expressed in only a few number of MLC (green in **B**), and ACTA1 (red in **C**) positive cardiomyocytes (arrows). **(D)** is the merged panel. Scale bar = 50 μm.

**FIGURE 8:**
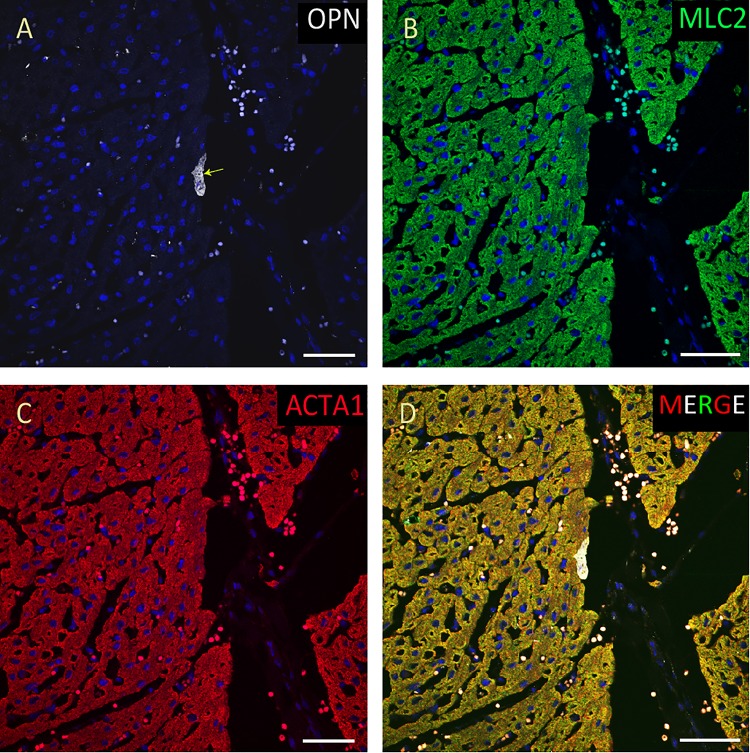
OPN expression is minimal in the cardiomyocytes from a pediatric patient without any infection. In the pediatric patient with ompholocele and without any infection, OPN (white in **A**) is barely expressed in a few number of MLC (green in **B**), and ACTA1 (red in **C**) positive cardiomyocytes (arrow). **(D)** is the merged panel. Scale bar = 50 μm.

**FIGURE 9:**
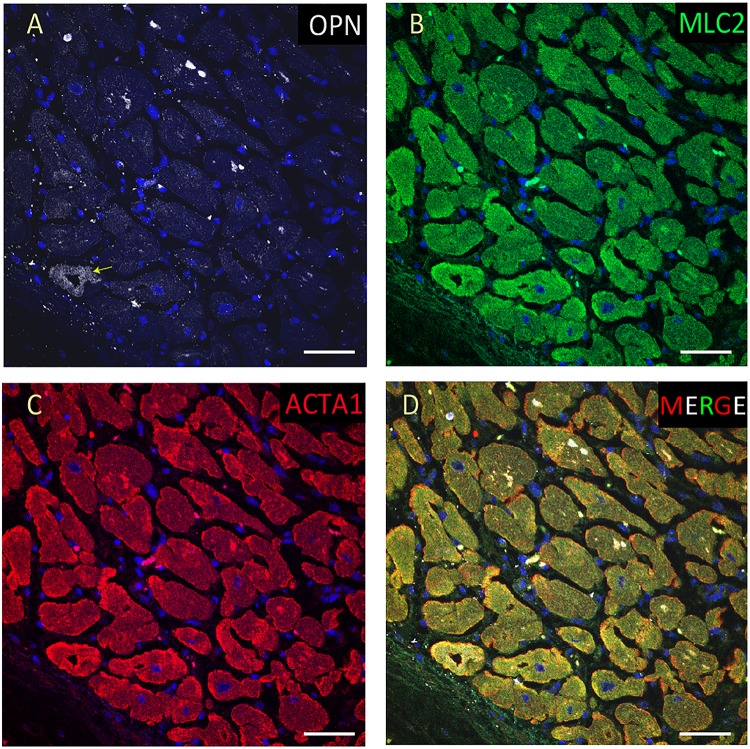
OPN expression is barely detected in the cardiomyocytes from an elderly patient with hypertension and sepsis. In the adult patient with sepsis and hypertension, OPN (white in **A**) is barely expressed in a few number of MLC (green in **B**), and ACTA1 (red in **C**) positive cardiomyocytes (arrow). **(D)** is the merged panel. Scale bar = 50 μm.

Immunofluorescence was used to investigate the OPN expression in paraffin-embedded sections. Samples were incubated for 45 min at 70 degrees, then dewaxed with two 5-min xylene washes and hydrated by 3-min graded ethanol washes of 100% (twice), 95%, 80%, and 70% followed by two 4-min water immersions. Antigen retrieval was then done by steaming the slides for 75 min in 1X Citrate Antigen Retrieval Buffer (Ab93678). Samples were rinsed with PBS then permeabilized with 0.2% Triton X-100 (Sigma-Aldrich) in PBS for 30 min, followed by blocking with 10% Donkey serum in TBST for 45 min. The samples were then incubated overnight at 4°C with three primary antibodies, Anti-Alpha Skeletal Muscle Actin, Alpha-Sr 1 (ACTA-1) (Abcam Ab28052; dilution 1:200), anti-hOPN (R&D AF1433; dilution 1:50), and anti-Myosin Light Chain (MLC2) (Abcam Ab92721; dilution 1:200) in blocking solution. ACTA-1 and MLC2 were used as cardiomyocyte markers. The following day the slides were washed with PBS then incubated for 1 h at room temperature with conjugated secondary antibodies Donkey anti-Mouse IgG, Alexa Fluor 568 (ThermoFisher Scientific A-10037; 1:400), Donkey anti-Goat IGG, Alexa Fluor 647 (ThermoFisher Scientific A-21447, 1:400), and Donkey anti-Rabbit IgG, Alexa Fluor 488 (ThermoFisher Scientific A-21206; 1:400) in blocking solution. DAPI was used to stain nuclei before mounting with ProLong Gold Antifade (Invitrogen P36934) and coverslips. After 2 days, the slides were scanned at 20 × magnification using the Olympus VS120–L100 Virtual Slide Microscope (Tokyo, Japan). At least five slides per specimen were stained to confirm results. Representative confocal images (shown in Figures 1–9) were captured at 40x magnification and 0.6x digital zoom using Z-stacking on a Zeiss LSM710 confocal microscope.

### Image Analyses and Quantification of OPN Positive Myocytes

All immunostaining scanned images were analyzed using Olympus OlyVIA 2.9 software. A quantitative analysis was performed independently by two investigators to manually count the number of ACTA1 and MLC2 positive cardiomyocytes that express OPN in representative fields. Five images per sample were counted and averaged.

## Results

To quantify the OPN protein expression, immunofluorescence was performed in paraffin-embedded sections of myocardial biopsies obtained from patients with different conditions (described in Table 1). All myocardial tissues in addition to two control kidney tissues were stained for OPN. As expected, the pediatric diseased kidney had a very high expression of OPN in the renal tubules (Figure 1A) and the adult healthy kidney had a moderate expression of OPN in the renal tubules (Figure 1B). All myocardial tissues were additionally co-stained with the myocyte markers ACTA1 and MLC2 to unequivocally identify the OPN positive cardiomyocytes.

**FIGURE 10:**
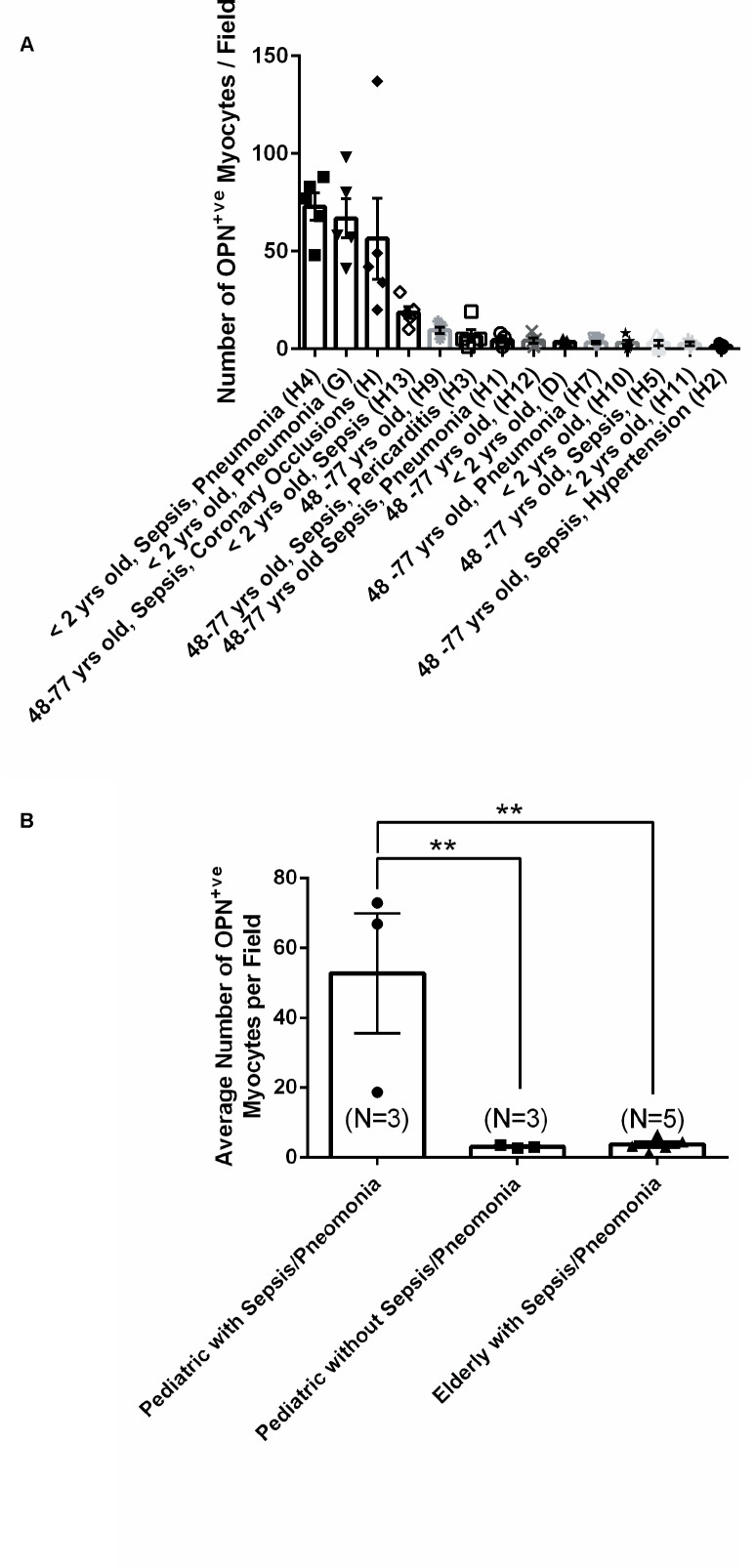
Quantification of the number of OPN positive cardiomyocytes in each of the fourteen myocardial specimens. **(A)** A quantitative analysis was performed to manually count the number of ACTA1 and MLC2 positive cardiomyocytes that express OPN in representative fields. Five images per sample were counted and averaged. **(B)** Statistical analysis was performed on the three main groups. *N* = 3–5 patients per group. ^∗∗^*P* < 0.001 using ANOVA with Tukey multiple correction.

From the 14 myocardial tissues analyzed, the three pediatric hearts with sepsis and/or pneumonia, showed the highest expression of OPN (Figures 2, 3, 5). Except for the heart from the old patient with sepsis and several severe coronary occlusions (Figure 4), all the other hearts from the pediatric patients and the mid-age and old patients had barely had any OPN expression in the cardiomyocytes (Figures 6–9). The microscopic images (Figures 1–9) are presented in the order of high to low expression of OPN. The quantification of the number of OPN positive cardiomyocytes in each of the fourteen myocardial tissues and in the three groups (pediatric with or without sepsis/pneumonia and elderly with sepsis/pneumonia) is shown in Figure 10.

## Discussion

Our study shows that OPN expression was increased in cardiomyocytes of pediatric patients with sepsis or pneumonia but not in those of elderly patients without evidence of comorbid Coronary Artery Disease. According to the Center for Disease Control and Prevention, in the United States 270,000 people die from sepsis each year (Rhee et al., 2017). For newborns sepsis was the seventh leading cause of infant death in 2016 (Kochanek et al., 2017), followed by influenza and pneumonia (Kochanek et al., 2017). In sepsis, respiratory tract infections, particularly pneumonia, are the most common and associated with the highest mortality (Mayr et al., 2014). Both sepsis and pneumonia have been reported to induce heart dysfunction (Merx and Weber, 2007; Rudiger and Singer, 2007, 2013; Corrales-Medina et al., 2011; Brown et al., 2014).

Myocardial dysfunction during sepsis and pneumonia has been variously associated with increased circulating cytokines and prostanoids, endothelin upregulation, impaired oxygen utilization, and increased levels of C-reactive protein (Kumar et al., 1996; Merx and Weber, 2007; Musher et al., 2007; Hunter and Doddi, 2010; Singanayagam et al., 2012). Increased OPN levels in plasma have also been reported (Kadota et al., 2005; Vaschetto et al., 2008), but the corresponding levels in cardiac tissues have not been investigated. OPN is overexpressed in the heart coincident with myocardial infarction (Trueblood et al., 2001), hypertrophy (Graf et al., 1997; Li et al., 2017), and myocarditis (Szalay et al., 2009) in a cell type- and stimulant-dependent manner (Singh et al., 2010). Mechanistically, interleukin-1β combined with interferon–γ (IFN-γ) increase OPN expression in cardiac microvascular endothelial cells (Singh et al., 1995), while angiotensin II alone or combined with TNF-α or interleukin-1β increase OPN gene expression in cardiac fibroblast (Xie et al., 2004), and glucocorticoid hormone can increase the expression of OPN in cardiomyocytes and cardiac microvascular endothelial cells (Singh et al., 1995).

Graf et al. (1997) showed that endothelin-1 and norepinephrine induce OPN expression in cultured rat neonatal cardiac myocytes. Interestingly high levels of circulating endothelin-1 have also been linked with sepsis (Bocking et al., 1979; Shindo et al., 1998; Rudiger and Singer, 2007), providing a possible explanation for our results in the pediatric specimens. In our studies the highest cardiomyocyte OPN levels were found in patient (H4), a baby born prematurely who died due to necrotizing enterocolitis. The subject had edematous heart muscle due sepsis and multi-organ failure, and patient (G), a child with fungal pneumonia. In both subjects the increased cardiomyocyte OPN expression may be linked with coincident inflammation. Our results are consistent with a central role for infection/inflammation in the activation of OPN expression in the pediatric heart. Notably, the children with full term ompholocele, holoprosencephaly, or premature birth, but no infection, showed only low-level cardiomyocyte OPN.

Interestingly, in the hearts of the older patients with sepsis, few cardiomyocytes expressed OPN. Among these, only the heart with severe coronary arterial occlusions showed high levels of cardiomyocyte OPN, consistent with the known link between Coronary Artery Disease, myocardial infarction, and OPN (Trueblood et al., 2001). It seems likely that age is a determining factor in the possible association between sepsis and cardiac OPN. Consistent with this, Luce et al. (2007) reported that cytokine responses differ widely between neonates and adults. Paliwal et al. (2012) reported higher OPN expression in macrophages of aged mice with skeletal muscle injury. Conversely, Schultz et al. (2002, 2004) reported exaggerated inflammatory responses in term and preterm infants compared to adults with attenuation of the pro-inflammatory reaction during infection that can lead to severe organ dysfunction in infants. Developing neonatal cardiomyocyte also differ from adults and there may be different responses to sepsis and inflammation (Luce et al., 2007).

Patients with pneumococcal pneumonia are at increased risk for concurrent acute cardiac events (Musher et al., 2007). Increased levels of inflammatory cytokines, C-reactive protein, and fibrinogen, along with increased myocardial demand for oxygen and lowered blood oxygen levels, are among the factors that have been reported to lead to cardiac dysfunction in patients with bacterial pneumonia. Based on our findings, increased OPN level may contribute to these pneumonia-caused cardiac events. However, further studies with larger sample sizes are needed to confirm relationships between cardiomyocyte OPN, inflammation, and age.

## Conclusion

In this study, we report markedly increased levels of OPN in cardiomyocytes of pediatric patients with sepsis or sepsis and pneumonia. Both sepsis and pneumonia involve activation of the immune system, inflammatory responses followed by cardiac dysfunction. This is the first study to report such increased cardiomyocyte OPN expression in pediatric sepsis or pneumonia. Our findings highlight a potentially important role for OPN in sepsis- or pneumonia-mediated cardiac dysfunction in pediatric patients.

## Ethics Statement

This study was carried out in accordance with the recommendations of the University of Miami Institutional Review Board (IRB protocol # 20180439). The study was exempt because the samples used were all autopsy samples.

## Author Contributions

CI, KP, and KJ-W performed the experiments. CI and KP analyzed the results. SG provided the pathology samples, clinical information, and data interpretation. CI and LS wrote the manuscript.

## Conflict of Interest Statement

The authors declare that the research was conducted in the absence of any commercial or financial relationships that could be construed as a potential conflict of interest.

## References

[B1] Ashizawa N. Graf K. Do Y. S. Nunohiro T. Giachelli C. M. Meehan W. P. (1996). Osteopontin is produced by rat cardiac fibroblasts and mediates A(II)-induced DNA synthesis and collagen gel contraction. *J. Clin. Invest.* 98 2218–2227. 10.1172/Jci119031 8941637 PMC507670

[B2] Bocking J. K. Sibbald W. J. Holliday R. L. Scott S. Viidik T. (1979). Plasma catecholamine levels and pulmonary dysfunction in sepsis. *Surg. Gynecol. Obstet.* 148 715–719. 432784

[B3] Brown A. O. Mann B. Gao G. Hankins J. S. Humann J. Giardina J. (2014). *Streptococcus pneumoniae* translocates into the myocardium and forms unique microlesions that disrupt cardiac function. *PLoS Pathog.* 10:e1004383. 10.1371/journal.ppat.1004383 25232870 PMC4169480

[B4] Chang J. H. Hung W. Y. Bai K. J. Yang S. F. Chien M. H. (2016). Utility of plasma osteopontin levels in management of community-acquired pneumonia. *Int. J. Med. Sci.* 13 673–679. 10.7150/ijms.16175 27647996 PMC5027185

[B5] Cobbs A. Ballou K. Chen X. George J. Zhao X. (2018). Saturated fatty acids bound to albumin enhance osteopontin expression and cleavage in renal proximal tubular cells. *Int. J. Physiol. Pathophysiol. Pharmacol.* 10 29–38. 29593848 PMC5871627

[B6] Corrales-Medina V. F. Musher D. M. Wells G. A. Chirinos J. A. Chen L. Fine M. J. (2012). Cardiac complications in patients with community-acquired pneumonia: incidence, timing, risk factors, and association with short-term mortality. *Circulation* 125 773–781. 10.1161/CIRCULATIONAHA.111.040766 22219349

[B7] Corrales-Medina V. F. Suh K. N. Rose G. Chirinos J. A. Doucette S. Cameron D. W. (2011). Cardiac complications in patients with community-acquired pneumonia: a systematic review and meta-analysis of observational studies. *PLoS Med.* 8:e1001048. 10.1371/journal.pmed.1001048 21738449 PMC3125176

[B8] Dal-Secco D. DalBó S. Lautherbach N. E. S. Gava F. N. Celes M. R. N. Benedet P. O. (2017). Cardiac hyporesponsiveness in severe sepsis is associated with nitric oxide-dependent activation of G protein receptor kinase. *Am. J. Physiol. Heart Circ. Physiol.* 313 H149–H163. 10.1152/ajpheart.00052.2016 28526706

[B9] Dhainaut J. F. Lanore J. J. de Gournay J. M. Huyghebaert M. F. Brunet F. Villemant D. (1988). Right ventricular dysfunction in patients with septic shock. *Intensive Care Med.* 14(Suppl. 2), 488–491. 10.1007/BF00256967 3403793

[B10] Ding W. Yousefi K. Goncalves S. Goldstein B. J. Sabater A. L. Kloosterboer A. (2018). Osteopontin deficiency ameliorates Alport pathology by preventing tubular metabolic deficits. *JCI Insight* 10.1172/jci.insight.94818 [Epub ahead of print]. 29563333 PMC5926939

[B11] Fortis S. Khadaroo R. G. Haitsma J. J. Zhang H. (2015). Osteopontin is associated with inflammation and mortality in a mouse model of polymicrobial sepsis. *Acta Anaesthesiol. Scand.* 59 170–175. 10.1111/aas.12422 25328143 PMC4936904

[B12] Graf K. Do Y. S. Ashizawa N. Meehan W. P. Giachelli C. M. Marboe C. C. (1997). Myocardial osteopontin expression is associated with left ventricular hypertrophy. *Circulation* 96 3063–3071. 10.1161/01.CIR.96.9.3063 9386176

[B13] Hunter J. D. Doddi M. (2010). Sepsis and the heart. *Br. J. Anaesth.* 104 3–11. 10.1093/bja/aep339 19939836

[B14] Icer M. A. Gezmen-Karadag M. (2018). The multiple functions and mechanisms of osteopontin. *Clin Biochem.* 59 17–24. 10.1016/j.clinbiochem.2018.07.003 30003880

[B15] Jardin F. Fourme T. Page B. Loubieres Y. Vieillard-Baron A. Beauchet A. (1999). Persistent preload defect in severe sepsis despite fluid loading: a longitudinal echocardiographic study in patients with septic shock. *Chest* 116 1354–1359. 10.1378/chest.116.5.1354 10559099

[B16] Jin X. Fu G.-X. Li X.-D. Zhu D.-L. Gao P.-J. (2011). Expression and Function of Osteopontin in Vascular Adventitial Fibroblasts and Pathological Vascular Remodeling. *PLoS One* 6:e23558. 10.1371/journal.pone.0023558 21949681 PMC3176202

[B17] Kadota J. Mizunoe S. Mito K. Mukae H. Yoshioka S. Kawakami K. (2005). High plasma concentrations of osteopontin in patients with interstitial pneumonia. *Respir Med.* 99 111–117. 10.1016/j.rmed.2004.04.018 15672859

[B18] Kato A. Okura T. Hamada C. Miyoshi S. Katayama H. Higaki J. (2014). Cell Stress Induces Upregulation of Osteopontin via the ERK Pathway in Type II Alveolar Epithelial Cells. *PLoS One* 9:e100106. 10.1371/journal.pone.0100106 24963635 PMC4070890

[B19] Khadour F. H. Panas D. Ferdinandy P. Schulze C. Csont T. Lalu M. M. (2002). Enhanced NO and superoxide generation in dysfunctional hearts from endotoxemic rats. *Am. J. Physiol. Heart Circ. Physiol.* 283 H1108–H1115. 10.1152/ajpheart.00549.2001 12181141

[B20] Kochanek K. D. Murphy S. Xu J. Arias E. (2017). Mortality in the United States, 2016. *NCHS Data Brief* 293 1–8. 29319473

[B21] Kumar A. Thota V. Dee L. Olson J. Uretz E. Parrillo J. E. (1996). Tumor necrosis factor alpha and interleukin 1beta are responsible for in vitro myocardial cell depression induced by human septic shock serum. *J. Exp. Med.* 183 949–958. 10.1084/jem.183.3.949 8642298 PMC2192364

[B22] Landesberg G. Gilon D. Meroz Y. Georgieva M. Levin P. D. Goodman S. (2012). Diastolic dysfunction and mortality in severe sepsis and septic shock. *Eur. Heart J.* 33 895–903. 10.1093/eurheartj/ehr351 21911341 PMC3345552

[B23] le Roux D. M. Zar H. J. (2017). Community-acquired pneumonia in children - a changing spectrum of disease. *Pediatr. Radiol.* 47 1392–1398. 10.1007/s00247-017-3827-8 29043417 PMC5608782

[B24] Leibovici L. Gafter-Gvili A. Paul M. Almanasreh N. Tacconelli E. Andreassen S. (2007). Relative tachycardia in patients with sepsis: an independent risk factor for mortality. *QJM* 100 629–634. 10.1093/qjmed/hcm074 17846061

[B25] Li J. Yousefi K. Ding W. Singh J. Shehadeh L. A. (2017). Osteopontin RNA aptamer can prevent and reverse pressure overload-induced heart failure. *Cardiovasc. Res.* 113 633–643. 10.1093/cvr/cvx016 28453726 PMC7526752

[B26] Liu Y. C. Yu M. M. Shou S. T. Chai Y. F. (2017). Sepsis-induced cardiomyopathy: mechanisms and treatments. *Front. Immunol.* 8:1021. 10.3389/fimmu.2017.01021 28970829 PMC5609588

[B27] Lopez B. Gonzalez A. Lindner D. Westermann D. Ravassa S. Beaumont J. (2013). Osteopontin-mediated myocardial fibrosis in heart failure: a role for lysyl oxidase? *Cardiovasc. Res.* 99 111–120. 10.1093/cvr/cvt100 23619422

[B28] Lorenzen J. M. Schauerte C. Hubner A. Kolling M. Martino F. Scherf K. (2015). Osteopontin is indispensible for AP1-mediated angiotensin II-related miR-21 transcription during cardiac fibrosis. *Eur. Heart J.* 36 2184–2196. 10.1093/eurheartj/ehv109 25898844 PMC4543785

[B29] Luce W. A. Hoffman T. M. Bauer J. A. (2007). Bench-to-bedside review: developmental influences on the mechanisms, treatment and outcomes of cardiovascular dysfunction in neonatal versus adult sepsis. *Crit. Care* 11:228. 10.1186/cc6091 17903309 PMC2556733

[B30] Lund S. A. Giachelli C. M. Scatena M. (2009). The role of osteopontin in inflammatory processes. *J. Cell Commun. Signal.* 3 311–322. 10.1007/s12079-009-0068-0 19798593 PMC2778587

[B31] Matkovich S. J. Al Khiami B. Efimov I. R. Evans S. Vader J. Jain A. (2017). Widespread down-regulation of cardiac mitochondrial and sarcomeric genes in patients with sepsis. *Crit. Care Med.* 45 407–414. 10.1097/CCM.0000000000002207 28067713 PMC5315660

[B32] Mattila J. T. Fine M. J. Limper A. H. Murray P. R. Chen B. B. Lin P. L. (2014). Pneumonia. Treatment and diagnosis. *Ann. Am. Thorac. Soc.* 11(Suppl. 4), S189–S192. 10.1513/AnnalsATS.201401-027PL 25148424 PMC5473649

[B33] Mayr F. B. Yende S. Angus D. C. (2014). Epidemiology of severe sepsis. *Virulence* 5 4–11. 10.4161/viru.27372 24335434 PMC3916382

[B34] Merx M. W. Weber C. (2007). Sepsis and the heart. *Circulation* 116 793–802. 10.1161/CIRCULATIONAHA.106.678359 17698745

[B35] Musher D. M. Rueda A. M. Kaka A. S. Mapara S. M. (2007). The association between pneumococcal pneumonia and acute cardiac events. *Clin. Infect. Dis.* 45 158–165. 10.1086/518849 17578773

[B36] Paliwal P. Pishesha N. Wijaya D. Conboy I. M. (2012). Age dependent increase in the levels of osteopontin inhibits skeletal muscle regeneration. *Aging* 4 553–566. 10.18632/aging.100477 22915705 PMC3461343

[B37] Parker M. M. McCarthy K. E. Ognibene F. P. Parrillo J. E. (1990). Right ventricular dysfunction and dilatation, similar to left ventricular changes, characterize the cardiac depression of septic shock in humans. *Chest* 97 126–131. 10.1378/chest.97.1.126 2295231

[B38] Poelaert J. Declerck C. Vogelaers D. Colardyn F. Visser C. A. (1997). Left ventricular systolic and diastolic function in septic shock. *Intensive Care Med.* 23 553–560. 10.1007/s001340050372 9201528

[B39] Qing C. Peishun S. Liying Z. Chunliang X. Chunxing Z. Yanyan H. (2014). An osteopontin-integrin interaction plays a critical role in directing adipogenesis and osteogenesis by mesenchymal stem cells. *Stem Cells* 32 327–337. 10.1002/stem.1567 24123709 PMC3961005

[B40] Rhee C. Dantes R. Epstein L. Murphy D. J. Seymour C. W. Iwashyna T. J. (2017). Incidence and Trends of Sepsis in US Hospitals Using Clinical vs Claims Data, 2009-2014. *JAMA* 318 1241–1249. 10.1001/jama.2017.13836 28903154 PMC5710396

[B41] Rudiger A. Singer M. (2007). Mechanisms of sepsis-induced cardiac dysfunction. *Crit. Care Med.* 35 1599–1608. 10.1097/01.CCM.0000266683.64081.02 17452940

[B42] Rudiger A. Singer M. (2013). The heart in sepsis: from basic mechanisms to clinical management. *Curr. Vasc. Pharmacol.* 11 187–195. 23506497

[B43] Schultz C. Rott C. Temming P. Schlenke P. Möller J. C. Bucsky P. (2002). Enhanced interleukin-6 and interleukin-8 synthesis in term and preterm infants. *Pediatr. Res.* 51:317. 10.1203/00006450-200203000-00009 11861936

[B44] Schultz C. Temming P. Bucsky P. GÖPel W. Strunk T. HÄRtel C. (2004). Immature anti-inflammatory response in neonates. *Clin. Exp. Immunol.* 135 130–136. 10.1111/j.1365-2249.2004.02313.x 14678274 PMC1808915

[B45] Shindo T. Kurihara H. Kurihara Y. Morita H. Yazaki Y. (1998). Upregulation of endothelin-1 and adrenomedullin gene expression in the mouse endotoxin shock model. *J. Cardiovasc. Pharmacol.* 31(Suppl. 1), S541–S544. 10.1097/00005344-199800001-00156 9595537

[B46] Singanayagam A. Singanayagam A. Elder D. H. Chalmers J. D. (2012). Is community-acquired pneumonia an independent risk factor for cardiovascular disease? *Eur. Respir. J.* 39 187–196. 10.1183/09031936.00049111 21737556

[B47] Singh K. Balligand J. L. Fischer T. A. Smith T. W. Kelly R. A. (1995). Glucocorticoids increase osteopontin expression in cardiac myocytes and microvascular endothelial cells. Role in regulation of inducible nitric oxide synthase. *J. Biol. Chem.* 270 28471–28478. 10.1074/jbc.270.47.28471 7499354

[B48] Singh M. Dalal S. Singh K. (2014). Osteopontin: at the cross-roads of myocyte survival and myocardial function. *Life Sci.* 118 1–6. 10.1016/j.lfs.2014.09.014 25265596 PMC4254317

[B49] Singh M. Foster C. R. Dalal S. Singh K. (2010). Osteopontin: role in extracellular matrix deposition and myocardial remodeling post-MI. *J. Mol. Cell Cardiol.* 48 538–543. 10.1016/j.yjmcc.2009.06.015 19573532 PMC2823840

[B50] Sodhi C. P. Phadke S. A. Batlle D. Sahai A. (2001). Hypoxia stimulates osteopontin expression and proliferation of cultured vascular smooth muscle cells. Potentiation by high glucose. *Diabetes* 50 1482–1490. 10.2337/diabetes.50.6.1482 11375351

[B51] Stawowy P. Blaschke F. Pfautsch P. Goetze S. Lippek F. Wollert-Wulf B. (2002). Increased myocardial expression of osteopontin in patients with advanced heart failure. *Eur. J. Heart Fail.* 4 139–146. 10.1016/S1388-9842(01)00237-9 11959041

[B52] Suffredini A. F. Fromm R. E. Parker M. M. Brenner M. Kovacs J. A. Wesley R. A. (1989). The cardiovascular response of normal humans to the administration of endotoxin. *N. Engl. J. Med.* 321 280–287. 10.1056/NEJM198908033210503 2664516

[B53] Szalay G. Sauter M. Haberland M. Zuegel U. Steinmeyer A. Kandolf R. (2009). Osteopontin: a fibrosis-related marker molecule in cardiac remodeling of enterovirus myocarditis in the susceptible host. *Circ. Res.* 104 851–859. 10.1161/CIRCRESAHA.109.193805 19246678

[B54] Tavener S. A. Long E. M. Robbins S. M. McRae K. M. Van Remmen H. Kubes P. (2004). Immune cell Toll-like receptor 4 is required for cardiac myocyte impairment during endotoxemia. *Circ. Res.* 95 700–707. 10.1161/01.RES.0000144175.70140.8c 15358664

[B55] Trueblood N. A. Xie Z. Communal C. Sam F. Ngoy S. Liaw L. (2001). Exaggerated left ventricular dilation and reduced collagen deposition after myocardial infarction in mice lacking osteopontin. *Circ. Res.* 88 1080–1087. 10.1161/hh1001.090842 11375279

[B56] van der Windt G. J. Hoogendijk A. J. Schouten M. Hommes T. J. de Vos A. F. Florquin S. (2011). Osteopontin impairs host defense during pneumococcal pneumonia. *J. Infect. Dis.* 203 1850–1858. 10.1093/infdis/jir185 21606543

[B57] Vaschetto R. Nicola S. Olivieri C. Boggio E. Piccolella F. Mesturini R. (2008). Serum levels of osteopontin are increased in SIRS and sepsis. *Intensive Care Med.* 34 2176–2184. 10.1007/s00134-008-1268-4 18807011

[B58] Vincent J. L. Bakker J. Marecaux G. Schandene L. Kahn R. J. Dupont E. (1992). Administration of anti-TNF antibody improves left ventricular function in septic shock patients. Results of a pilot study. *Chest* 101 810–815. 10.1378/chest.101.3.810 1541150

[B59] Watts J. A. Kline J. A. Thornton L. R. Grattan R. M. Brar S. S. (2004). Metabolic dysfunction and depletion of mitochondria in hearts of septic rats. *J. Mol. Cell Cardiol.* 36 141–150. 10.1016/j.yjmcc.2003.10.015 14734056

[B60] Xie Z. Singh M. Singh K. (2004). ERK1/2 and JNKs, but not p38 kinase, are involved in reactive oxygen species-mediated induction of osteopontin gene expression by angiotensin II and interleukin-1beta in adult rat cardiac fibroblasts. *J. Cell. Physiol.* 198 399–407. 10.1002/jcp.10419 14755545

[B61] Zhang Z.-X. Shek K. Wang S. Huang X. Lau A. Yin Z. (2010). Osteopontin expressed in tubular epithelial cells regulates NK cell-mediated kidney ischemia reperfusion injury. *J. Immunol.* 185 967–973. 10.4049/jimmunol.0903245 20548025

[B62] Zhong J. Hwang T. C. Adams H. R. Rubin L. J. (1997). Reduced L-type calcium current in ventricular myocytes from endotoxemic guinea pigs. *Am. J. Physiol.* 273(5 Pt 2), H2312–H2324. 10.1152/ajpheart.1997.273.5.H2312 9374768

